# Myths and Methodologies: Standardisation in human physiology research—should we control the controllables?

**DOI:** 10.1113/EP091557

**Published:** 2024-05-19

**Authors:** Lucy H. Merrell, Oliver J. Perkin, Louise Bradshaw, Harrison D. Collier‐Bain, Adam J. Collins, Sophie Davies, Rachel Eddy, James A. Hickman, Anna P. Nicholas, Daniel Rees, Bruno Spellanzon, Lewis J. James, Alannah K. A. McKay, Harry A. Smith, James E. Turner, Francoise Koumanov, Jennifer Maher, Dylan Thompson, Javier T. Gonzalez, James A. Betts

**Affiliations:** ^1^ Centre for Nutrition, Exercise and Metabolism University of Bath Bath UK; ^2^ National Centre for Sport and Exercise Medicine, School of Sport, Exercise and Health Sciences Loughborough University Loughborough UK; ^3^ Mary MacKillop Institute for Health Research Australian Catholic University Melbourne Australia; ^4^ School of Sport, Exercise and Rehabilitation Sciences University of Birmingham Birmingham UK

**Keywords:** control, physiology, standardisation

## Abstract

The premise of research in human physiology is to explore a multifaceted system whilst identifying one or a few outcomes of interest. Therefore, the control of potentially confounding variables requires careful thought regarding the extent of control and complexity of standardisation. One common factor to control prior to testing is diet, as food and fluid provision may deviate from participants’ habitual diets, yet a self‐report and replication method can be flawed by under‐reporting. Researchers may also need to consider standardisation of physical activity, whether it be through familiarisation trials, wash‐out periods, or guidance on levels of physical activity to be achieved before trials. In terms of pharmacological agents, the ethical implications of standardisation require researchers to carefully consider how medications, caffeine consumption and oral contraceptive prescriptions may affect the study. For research in females, it should be considered whether standardisation between‐ or within‐participants in regards to menstrual cycle phase is most relevant. The timing of measurements relative to various other daily events is relevant to all physiological research and so it can be important to standardise *when* measurements are made. This review summarises the areas of standardisation which we hope will be considered useful to anyone involved in human physiology research, including when and how one can apply standardisation to various contexts.

## 1 INTRODUCTION

**FIGURE 1:**
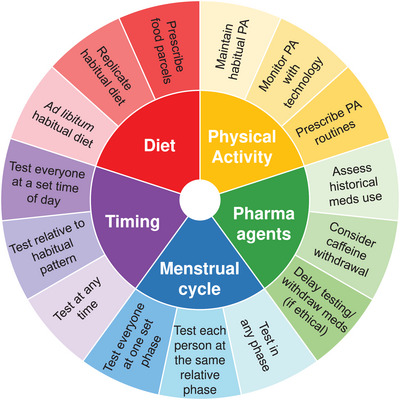
A summary of key variables to consider controlling when designing studies of human physiology, with examples to demonstrate continuums of practical strategies that may be employed from little control to tight control. The degree of control for each variable within a given study should be specific to the context of the research question and practical constraints. Meds, medications; PA, physical activity.

When conducting physiology research that involves the testing of human participants, there can be a seemingly endless number of methodological decisions related to experimental design, sampling/recruitment, protocol and analysis. One particularly important aspect to consider is the standardisation or control of extraneous/confounding variables, which can help to more confidently isolate the relationships between the independent and dependent variables of interest. Typical behavioural and biological factors that can vary naturally over time and may benefit from some degree of pre‐test standardisation include variability in lifestyle (e.g., diet and activity) or natural rhythms in metabolism (e.g., menstrual or circadian). This paper will consider just some of these key variables that may benefit from standardisation in human physiology research. Whilst it is impossible to cover an exhaustive list of what to standardise in any study (or whether that standardisation has indeed been successful), we have attempted to address those variables that have the greatest potential to confound a study, have broadest relevance to a variety of fields, and/or provide novel insights that are not commonly discussed within the literature (see Figure 1).

In terms of scope, this review will be relevant to research that involves the recruitment and testing of human participants but will focus particularly on factors that can vary over time within each individual (so can either be controlled to standardise conditions or can be allowed to vary but potentially monitored and adjusted for in analysis). By contrast, we will not be considering any stable personal characteristics that can be difficult to manipulate (control) ahead of testing, which instead tend to be accounted for in advance via study eligibility criteria. In each of the ensuing sections (i.e., food and fluid, physical activity, pharmacological agents, menstrual cycle, and other biological rhythms), we will consider the methods and relative merits of various pre‐trial controls and, in cases where standardisation is deemed appropriate, whether key variables should be matched within or between participants. As can be seen, the study context, population and resources hugely influence principles of standardisation, and therefore specific recommendations will not be made here. Instead, considerations of standardisation will be discussed to inform decisions relevant to your own research questions.

## 2 STANDARDISATION OF FOOD AND FLUID

Human physiology fundamentally seeks to understand how the human body responds to various stimuli, yet the response to any given stimulus tends to depend on the precise context in which measurements are made. Diet represents a prime example of this context, and there are numerous considerations when conducting human physiology research about whether diet should be controlled prior to measurements and, if so, the degree and time course of standardisation that may be warranted. Standardisation of dietary intake may be defined as ‘all methods of minimising pre‐existing differences in dietary intake or nutritional status of the participant’, the complexity of which is often overlooked yet varies widely in current literature (Jeacocke & Burke, 2010). It can encompass everything from no control whatsoever, to minimal control or complete control—as examples, researchers may: (1) allow all participants to eat freely (ad libitum); (2) simply provide guidance on what *not* to consume in a given time frame (e.g., avoid alcohol the day before testing); (3) provide guidance on *when* to eat/stop eating (e.g., arrive after a >10 h fast); (4) ask participants to weigh and record exactly what and when they eat, possibly with replication on any subsequent occasions; or (5) provide participants with all nutrition to consume over a given period.

Researchers should consider the intended use or translation of their findings to decide whether participants should be fasted or fed when completing experiments. Acute nutritional intake can influence systemic metabolite/hormone concentrations and fluxes, glycogen content, hydration status and hunger, which may result in priming effects persisting to subsequent meals (i.e., second‐meal effect). How long such carry‐over effects persist between meals may depend on the type of foods ingested, given that some nutrients elicit a more or less prolonged postprandial response than others (e.g., metabolic responses to glucose and protein ingestion (2–4 h; Atherton et al., 2010) are generally shorter than responses to dietary fats (3–6 h; Ruge et al., 2009)).

There are two broad approaches researchers can employ to control participants’ diets. The first is that researchers may allow participants to record and replicate habitual dietary intake, where any uneaten food can be weighed and adjustments made to subsequent trials. Ad libitum consumption may subsequently improve external validity, feasibility and practicality in the long‐term. However, self‐report methods of dietary intake are often limited by under‐reporting (34% lower on average relative to doubly labelled water; Bates et al., 2014) and rely on participant compliance in both recording and replication (Subar et al., 2015). Of course, experimental physiologists may rely on a basic cost–benefit analysis when choosing which protocols to implement (Jeacocke & Burke, 2010). Alternatively, researchers may provide participants with standardised meals prior to experimentation to attempt precise control over dietary intake, and perhaps reduce both between‐ and within‐participant variability (Kozior et al., 2022). Liquid or solid meals may be provided in fixed quantities and/or individualised to meet participants’ energy and nutritional requirements—for example, relative to body mass or metabolic requirements (El‐Chab et al., 2019). Food provision is common when diet may significantly impact physiology and when small expected effect sizes demand very precise results (El‐Chab et al., 2016). However, provided food will almost always deviate from a participant's usual diet, which may in fact introduce unintended consequences or increased variance in response.

In terms of fluid intake, alcoholic and other energy‐containing beverages (milk, carbonated beverages, *etc*.) are usually well‐controlled before trials as part of the general diet, whereas water and energy‐free beverages are often permitted ad libitum. Alcoholic beverages (at least in large amounts) have profound and long‐lasting effects on physiology (Schutz, 2000), and therefore need a very good reason not to be controlled. However, the common recommendation for participants to consume ∼500 mL water 1–2 h before trials (whilst adequate for control purposes) is unlikely to correct meaningful hypohydration or ‘ensure adequate hydration’, as many authors assert. Hydration status could also be measured, for example, using urine osmolality/creatinine, to correct for urinary variables for concentration effects, if deemed important to the variable(s) of interest, although it should be noted that differences may present if time since the last urination or fluid intake varies between conditions (Cheuvront et al., 2015). Given that relatively small alterations in water intake/balance can influence metabolism and performance (Bardis et al., 2013; Logan‐Sprenger et al., 2015), it may be important that all fluids are recorded alongside other foods/beverages.

Food/fluid intake is often administered without blinding (i.e., open label), meaning intake (or lack thereof) may induce placebo (or nocebo) effects. This may influence outcomes, particularly those with a behavioural or subjective component such as appetite ratings using visual analogue scales (VAS). For example, compared to consuming no breakfast, administering an energy‐free placebo or carbohydrate‐containing (1.5–2 g carbohydrate/kg) breakfast enhanced endurance (Mears et al., 2018) and strength (Naharudin et al., 2020) performance to a similar extent. It is therefore at the authors’ discretion to decide whether open administration of food/fluid in trials could influence outcomes beyond what is intended.

## 3 STANDARDISATION OF PHYSICAL ACTIVITY

Just as nutrition researchers are typically aware of the need to standardise participants’ diets prior to testing, it is second nature for exercise physiologists to attempt to standardise pre‐testing physical activity to minimise perturbations in physiological parameters during recovery from recent exercise bouts. However, the broad range of reasons to consider standardisation of physical activity may be less well appreciated in the wider field of physiology and beyond. For example, muscle glycogen stores may be depleted for up to 24 h after strenuous endurance exercise (Starling et al., 1997). This would certainly compromise exercise test performance, but may also impact postprandial insulin sensitivity (Jensen et al., 2011), lipid metabolism (Gill & Hardman, 2000), resting energy expenditure (Gillette et al., 1994) and even estimates of body composition by dual‐energy X‐ray absorptiometry (DXA; Bone et al., 2017), thus confounding inferences about metabolic health.

Akin to control of dietary factors, a continuum also exists for standardisation of physical activity. One common practice is to request that participants ‘maintain habitual physical activity’ and/or ‘refrain from strenuous exercise’ for 24 h before laboratory visits. Particularly for between‐participant comparisons, researchers may need to define exercise intensity in either relative or absolute terms, for example. Whilst the terminology may be open to interpretation, this approach should ensure sufficient time for recovery from previous exercise bouts for most metabolic measurements. It is advisable to avoid the complete removal of all daily physical activity (unless it is an inherent feature of the study design), as even 2 days of very sedentary behaviour (>14 h sitting/day) can increase postprandial lipaemia (Kim et al., 2016). Another strategy may be using modern technology to objectively assess physical activity prior to testing so that this complex behaviour can at least be confidently recorded and retrospectively accounted for in analysis (Thompson & Batterham, 2013), or even standardised between conditions in real‐time based on live feedback. Indeed, further standardisation procedures may be put in place depending on sensitivity of outcome measures to prior physical activity or exercise, for example, prescribing pre‐trial physical activity or exercise routines based on heart rate, but must be practicable for participants.

Standardisation of physical activity prior to testing is particularly important for intervention studies examining the enduring or cumulative effects of repeated acute stimuli (diet or exercise) to change phenotype. For example, whilst endurance‐trained individuals display greater insulin sensitivity and decreased postprandial lipaemia compared to inactive individuals, both these markers are significantly altered for up to 36 h after a single exercise bout in all populations (Horowitz, 2007). To measure chronic changes, a sufficient wash‐out interval following recent exercise may be necessary to avoid any confounding carry‐over effects from that recent activity (unless those acute effects of a recent bout are inherent to the research question).

For parallel group designs, standardisation procedures should ideally be consistent for all participants, including controls groups. However, regardless of study design, consideration should be given to the physiological heterogeneity introduced by habitual and/or lifelong physical activity behaviours, which may add noise to measurements and undermine inferences. For example, habitually sedentary individuals are more likely to have distinct metabolic responses to acute feeding experiments and may experience greater improvements in metabolic health during physical activity or diet interventions than highly active individuals. Where research is reliant on participants with a range of baseline characteristics, a minimisation strategy could be considered (Altman & Bland, 2005) and/or variables added in as a priority for randomisation.

Familiarisation with study procedures, particularly physical performance tests, is an important feature in study design and may warrant reflection when considering physical activity standardisation during study design. Firstly, appropriate familiarisation procedures can minimise learning effects during testing but researchers should be careful to balance the number of familiarisation trials to adequately reduce variability against the number of sessions that may induce training or learning effects (Mattocks et al., 2017). Of course, the extent of familiarisation required is highly variable between studies, owing to factors such as participant characteristics and research outcomes (Juntip & Pornpimol, 2021). Furthermore, subsequent experimental trials should occur after full recovery from familiarisation sessions but before any familiarisation effects have subsided.

## 4 STANDARDISATION OF PHARMACOLOGICAL AGENTS

Pre‐existing medical conditions sometimes warrant exclusion from research studies, but it is often in the interests of study generalisability that people with some diseases are represented—or indeed that disease is the primary focus of research. Many studies exclude people diagnosed with long‐term medical conditions, and most studies avoid making measurements on people with a current short‐term illness (e.g., upper respiratory tract infection). However, not all studies differentiate between participants who have—or have not had—a medical condition in the past, so‐long as the condition resolved within an arbitrary period (e.g., 10 years). However, past treatment of conditions, especially cancer, leaves a lasting fingerprint on physiology (Arana Echarri et al., 2023), and differences between individuals can even be detected whether they have previously encountered mild asymptomatic infections or not (e.g., *Cytomegalovirus*; Turner et al., 2010). While inclusion and exclusion of stable traits is beyond the scope of this paper, some medical conditions require participants to take pharmacological agents (i.e., medications) that may introduce bias, and therefore some degree of standardisation should be considered.

Two of the most common examples of prescribed and over‐the‐counter medications that are relevant to this discussion are antibiotics and non‐steroidal anti‐inflammatory drugs (NSAIDs). The former are typically prescribed for only short periods but even a single course of antibiotics can alter the gut microbiome (Zaura et al., 2015). Alterations to the gut microbiome could influence wider physiological responses for several months, so pre‐trial standardisation should allow sufficient time for the recovery of gut microbiota if that is likely to affect study outcomes. NSAIDs are commonly taken therapeutically for either short‐ or long‐term pain relief and can exert a wide range of effects, from suppressing muscle protein synthesis, to prolonging the time needed for recovery from exercise (Bateman et al., 2023; Lundberg & Howatson, 2018; Maseda & Ricciotti, 2020) and, like antibiotics, altering gut microbe composition (Maseda & Ricciotti, 2020). In research with older adults, control of medication may be difficult with age‐ and physical activity‐matched controls, as well as interactions with other pharmacological agents not necessarily of concern to study outcomes. Pre‐trial screening should therefore establish whether participants are taking any medications that could affect trial outcomes and consider whether it is possible to standardise the potential influence of that medication. This can sometimes be achieved either by delaying the research until the effects of medications have subsided or, if ethical, by delaying medications until the research is complete.

Beyond prescription or over‐the‐counter medications, another commonly consumed pharmacological agent with profound and wide‐reaching physiological effects is caffeine. It is common for research participants to be asked to abstain from caffeine consumption in the hours or days before a study, yet a review summarising 57 experimental studies on caffeine withdrawal found that 27% of participants showed caffeine withdrawal syndrome (i.e., headaches, decreased energy/alertness; Juliano & Griffiths, 2004). It is therefore debatable whether acute restriction of caffeine intake removes a potential confounding variable or introduces one.

Lastly, hormonal contraceptives are generally not included in the list of long‐term medications that prohibit participation in research studies, largely because so many women use this form of contraception. However, studies have shown that these types of drugs can exert effects on various physiological systems, for example, increasing markers of chronic low‐grade inflammation such as C‐reactive protein concentrations (Morin‐Papunen et al., 2008; Piltonen et al., 2012). The standardisation of contraceptive use in research may therefore take the same approaches as were proposed for the other medications described above or could be informed by considering the likely variability in outcomes at various stages in the menstrual cycle, as will be addressed in the next section.

## 5 STANDARDISATION OF MENSTRUAL CYCLE

**FIGURE 2:**
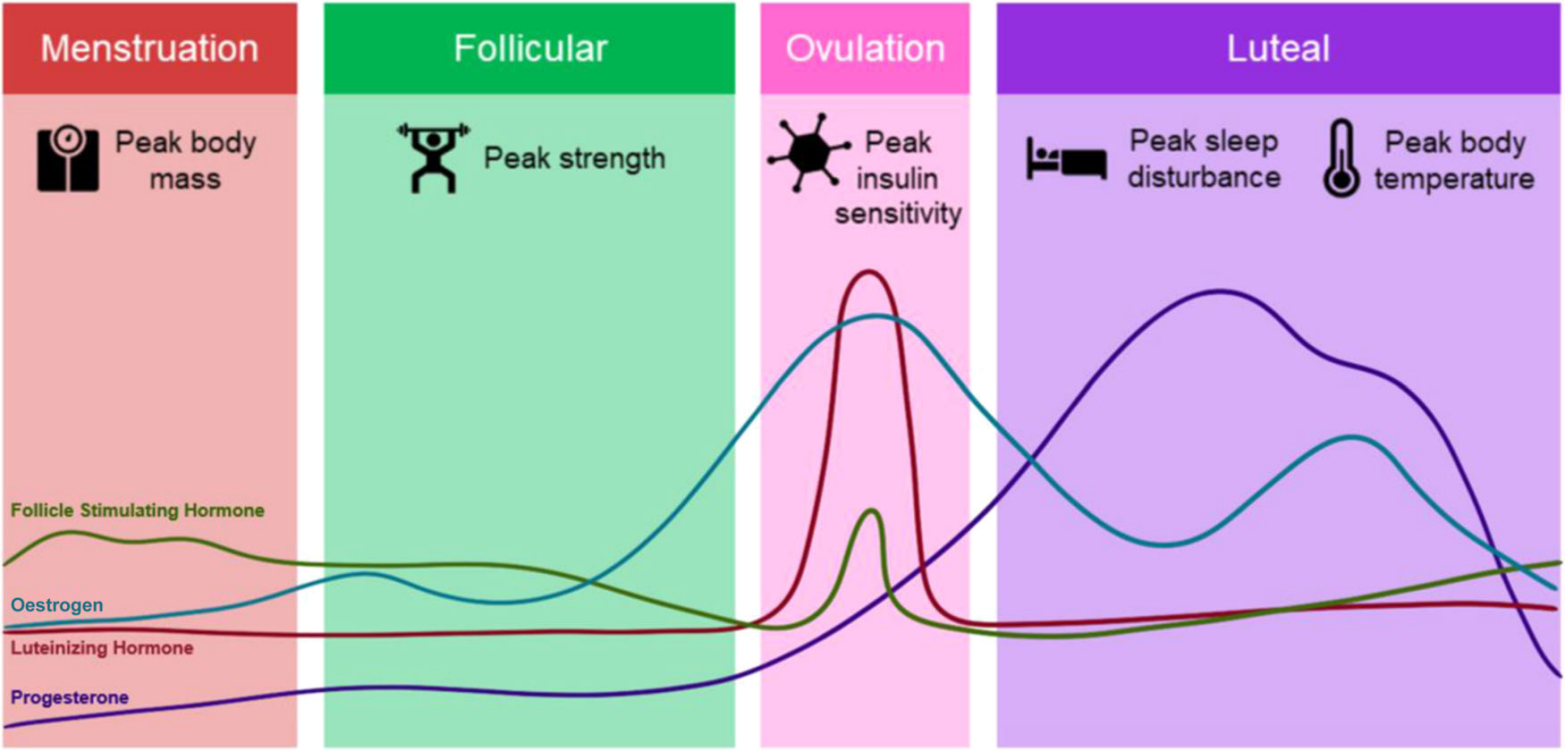
A graphical representation of the endogenous ovarian hormone fluctuation throughout a ‘typical’ menstrual cycle in eumenorrhoeic women. Potential phase outcome data derived from Baker and Driver (2007), Colenso‐Semple et al. (2023), Meendering et al. (2005), Oosthuyse and Bosch (2010).

The menstrual cycle is characterised by cyclic fluctuations in female sex hormones across a typical period of 21–35 days, leading to distinctly varied hormonal environments (Figure 2), with evidence that this variability can be linked to alterations in certain physiologically relevant outcomes.

Hormone profiles differ significantly depending on menstrual status (pre‐menarche, eumenorrhoeic, amenorrhoeic, peri‐menopausal, pregnant or post‐menopausal; Elliott‐Sale et al., 2021). If a study involves naturally menstruating or eumenorrhoeic females, standardisation of the menstrual cycle can occur either within‐ or between‐participants, depending on the study outcomes. Standardising within‐participants can occur by allowing a complete menstrual cycle washout period between trials to ensure participants are studied in the same phase relative to the first. Alternatively, between‐participant standardisation often involves identification of a specific phase and testing each participant in that same phase. Importantly, these approaches require logistical flexibility, as trial days need to be scheduled within a specific time frame.

Menstrual cycle phase should be tightly controlled for when the research question has a (sex) hormone‐driven hypothesis. For instance, outcomes such as lipid concentrations or resting metabolic rate can be affected by oestrogen concentration (Benton et al., 2020; Palmisano et al., 2018), and therefore testing at specific phases of the menstrual cycle may be important when conducting sequential measurements or looking for small changes. Measurement of systemic hormone concentrations, alongside the confirmation of ovulation with urinary luteinizing hormone, can be included in testing protocols to enhance confidence in the participants’ menstrual status/phase (Janse et al., 2019). However, participant burden and research costs may increase with the additional layer of methodological control, and outcomes may be less generalisable across other phases of the cycle or across women with different hormonal profiles. Alternatively, researchers may decide that not standardising menstrual cycle phase/status can reduce participant burden, and improve recruitment, retention and ecological validity. This approach may be preferable for large, free‐living cohort studies or research on niche populations where the available recruitment pool is limited. Regardless of the approach taken, researchers should consider comprehensively reporting menstrual characteristics of the female participants. This includes distinguishing eumenorrhoeic from naturally menstruating participants, describing cycle length and frequency, documenting hormonal contraception usage and diagnosed menstrual conditions (Elliott‐Sale et al., 2021).

## 6 STANDARDISATION OF TIMING

Whilst some of the other factors addressed above clearly have the potential to markedly influence physiological measurements, they are not always relevant to the standardisation of every study (i.e., male participants need not consider the menstrual cycle, not everyone is taking regular medication, and many people do not eat a very varied diet day‐to‐day). By contrast, every one of us is experiencing constant change via the passage of time from one minute to the next, so researchers cannot avoid the need to consider exactly *when* measurements will be made. Indeed, time is possibly the most investigated variable in all of science. The timing of physiological processes and measurements can be viewed in terms of both absolute time (e.g., hh:mm, date) and time relative to contextual patterns in the environment, metabolism and behaviour. Specifically, the mammalian circadian timing system can synchronise our biological rhythms with repeating cycles of light and darkness, waking and sleeping, and with transitions between the fasted‐ and fed‐state (Smith & Betts, 2022). Daily rhythms in systemic insulin and glucose concentrations are a well‐recognised example of this, with total plasma insulin secretion rates reported as being up to 50% higher in the afternoon compared to the morning (Poggiogalle et al., 2018). The precise time period when measurements are made should therefore be standardised or at least reported in human physiology research, since results may vary seasonally across the year and/or between different times of day. Moreover, underlying rhythms in metabolism and behaviour can be acutely disrupted by certain factors that should therefore be accounted for when designing experiments and scheduling participants (e.g., recent travel between different global time zones, artificial/nocturnal light exposure, sleep deprivation, unusual eating patterns).

In view of the above, it is common for many studies in human physiology to report an absolute, between‐participant method of time standardisation, for example: ‘all participants arrived at the laboratory at 08.00 ± 1 h’. Whilst this approach certainly has merits in terms of controlling for time of day and possibly therefore other directly associated environmental cues (e.g., natural daylight exposure), it may in fact introduce additional variance due to individual differences in the scheduling of other daily events. For example, one participant who usually wakes at 0900 h would commence testing when they would usually be asleep in bed, whereas another participant who usually wakes at 0500 h would have to wait to commence testing without starting their usual daily routine of eating and activities. Researchers may therefore do well to consider adopting relative, within‐participant methods of time standardisation—for example, measurements could be scheduled to start 1 h following each individual's usual time of waking, or so that meal/exercise tests commence at the time when each individual would usually eat or be active. Another approach may be to consider use of Critical Difference statistical modelling to quantify the extent of natural biological variation that may be due to interindividual variability in daily rhythms (Rose et al., 2018).

## 7 CONCLUSION

This review encourages researchers in human physiology to consider five key areas of standardisation: (1) food and fluid intake, (2) physical activity, (3) pharmacological agents, (4) menstrual cycle, and (5) timing. This piece is deliberately non‐prescriptive, providing researchers the freedom to reflect on the context, participants and resources of their research, and to make informed decisions where standardisation may, or may not, be beneficial. Consistent with that reasoning, Table 1 provides examples of research scenarios that consider standardisation strategies, with rationales for potential solutions; the table is not intended to represent a prescriptive list—only possible reasons for controlling certain factors given the context of those examples.

**TABLE 1:** Examples of common sources of variation and potential solutions/mitigations in reference to key areas of standardisation.

Area of standardisation	Typical research design context—key considerations	Potential approach—with supporting rationale based on context
Food	Assessments of tissue insulin sensitivity via hyperinsulinaemic–euglycaemic clamp E.g., *n* = 10 participants receive new drug versus placebo (cross‐over design) *Q: Whether to control diet 24 h before tests*?	Within‐participant standardisation could be justified in this context (i.e., each individual weighs and records their usual diet prior to the first test and replicates ahead of the second). This approach may be deemed appropriate on the basis that outcomes are incredibly precise and reactive (i.e., clamp), so employing no control (i.e., ad libitum diet) could introduce error from recent food intake. By contrast, prescribing a standardised diet to all participants (i.e., between‐participant standardisation) could be burdensome, may deviate from the habitual diet of some more than others, and could render the findings less generalisable (i.e., specific to the context of the prescribed diet)
Fluid	Assessments of perceived effort during prolonged running in the heat E.g., *n* = 25 runners from warmer climates versus *n* = 25 runners from cooler climates (parallel groups design) *Q: Whether to control pre‐test hydration*?	Between‐participant standardisation could be justified in this context (i.e., encourage a minimum fluid intake over the days prior to testing and have all fluid intake recorded). This approach may not fully standardise hydration status prior to testing but may minimise the probability of any marked hypohydration ahead of testing, which cannot typically be rectified by fluid ingestion on the day of testing
Fasting status	Assessments of micronutrient status after supplementation with a novel ingredient E.g., *n* = 100 participants supplement daily for 6 weeks with pre–post bloods (time‐series design) *Q: Whether to draw bloods before breakfast*?	Between‐participant standardisation for all participants to remain in an overnight fasted‐state may be justified in this context as the acute systemic response of micronutrients to the breakfast could mask subtle effects of the supplements. In addition, this approach avoids any between‐participant variance according to individuals’ habitual breakfast preferences, since all are fasted. Moreover, given that a novel ingredient is being tested, research could benefit first from establishing responses under more controlled conditions (proof‐of‐principle), then examine whether results vary under various specific fed‐states
Alcohol	Single assessment of glycaemic response to a new food E.g., *n* = 50 participants ingest the product and monitor postprandial glycaemic response (cross‐sectional design) *Q: Whether to control alcohol 24 h before tests*?	In this context, between‐participant standardisation for all participants to abstain from alcohol for at least 24 h may be justified on the basis that alcohol can elicit marked and persistent effects on glucose metabolism. Within‐participant standardisation is not relevant since there is only one condition, whereas no standardisation (i.e., permitting ad libitum alcohol intake) would introduce unnecessary uncertainty and potential systematic bias into the estimate of how much the product increases blood glucose (i.e., the primary research question)
Physical activity	Assessments of muscle glycogen during intermittent exercise after altitude training E.g., *n* = 20 athletes train at altitude versus *n* = 20 athletes train at sea level (parallel groups design) *Q: Whether to control PA 24 h before tests*?	Between‐participant standardisation may be deemed necessary to limit all strenuous physical activity including exercise (applicable to all participants 24 h prior to testing), but participants are free to continue with habitual PA within these constraints. Researchers may see value in objective quantification of PA (e.g., accelerometers) to monitor activity, potentially with a minimal prescription (e.g., one brisk walk) to reduce chances of the groups being systematically different
Medication	Assessment of stimulated immune response to infection following weight loss E.g., *n* = 200 obese males (>50 years) randomised to 3‐month lifestyle change versus control (parallel groups design) *Q: Whether to control medication before tests*?	Previous medical conditions and use of medications (past/present) could be assessed in relation to study outcomes and participants excluded if such factors are thought to be confounding. Routine medications may require closer consideration on a case‐by‐case basis, due to their widespread use in this population. For example, the number of participants in this case taking statins would limit recruitment if deemed ineligible, and asking participants to refrain from taking the medication would not only be impractical (and potentially unethical), but also limits the generalisability of the study to the population for whom the findings may be most valuable
Caffeine	Assessments of adipose tissue lipolysis in varied environmental temperatures E.g., *n* = 20 habitual caffeine consumers tested in hot versus cold ambient conditions (cross‐over design) *Q: Whether to control caffeine 24 h before tests*?	Within‐participant standardisation (ad libitum consumption) may be most appropriate here, which could be achieved by asking participants to keep a record of their caffeine consumption 24 h prior to testing. Asking all participants to refrain from consumption (between‐participant standardisation) may not be suitable for habitual caffeine consumers due to withdrawal symptoms becoming a potential confounder
Hormonal contraceptives	Single assessments of sleep quality and systemic endocrine status E.g., *n* = 500 pre‐menopausal women undergo polysomnography and a blood test for cortisol concentrations (cross‐sectional design) *Q: Whether to include hormonal contraceptive users*?	While both outcome measures may be affected by hormonal contraceptive use, the high prevalence of these pharmacological agents among premenopausal women may justify their inclusion. Using a mixed cohort of women who are naturally cycling and using one or many contraceptive formulations would increase the generalisability of findings to a wider population. Furthermore, the sample size here is sufficiently large that adequate power should still be achieved. For all women, menstrual characteristics such as life stage, hormonal contraceptive formulation, cycle lengths and frequency should be captured and reported where possible
Menstrual cycle	Assessments of appetite and energy intake with a digital wellness intervention E.g., *n* = 60 menstruating females complete meal tests following 4‐weeks’ control versus use of a mobile device application (time‐series design) *Q: Whether to control for menstrual cycle phase*?	In naturally cycling women, the design of a 4‐week intervention may permit testing broadly within the same phase for each condition (i.e., within‐participant standardisation, where participants are tested within the same relative phase). Of course, this is caveated by each woman's likely variation from a ‘textbook’ 28‐day cycle and therefore characterisation of cycle may be necessary prior to testing. Here, researchers could pair the analysis to reflect contrasts of all phases (e.g., high hormone (luteal) or low hormone (follicular) phases) and/or where stages of the cycle (and where possible, hormone concentrations) have been recorded, these may be used in *post‐hoc* analysis It is also worth considering that not all naturally cycling women will menstruate or have cyclical hormonal fluctuations (i.e., post‐menopausal, pregnancy, amenorrhea). Accordingly, if hormonal changes are thought to impact primary outcome measures, inclusion criteria may stipulate only women with a regular (9 or more periods per year, 21–35 days in length) menstrual cycle be included
Timing	Assessment of salivary cortisol concentrations according to chronotype E.g., *n* = 50 morning‐types and *n* = 50 evening‐types provide daily saliva samples (parallel groups design) *Q: When to collect the saliva samples*?	Within‐participant standardisation could be justified in this context, where participants are not tested at a set time of day (absolute time of day) but instead provide a sample at a time relative to their habitual time of waking (e.g., +1 h)

## AUTHOR CONTRIBUTIONS

Food and fluid section written by Lucy H. Merrell, Anna P. Nicholas, Lewis J. James and Sophie Davies; Physical activity section written by Oliver J. Perkin, Adam J. Collins and James A. Hickman; Pharmacological agents section written by James E. Turner, Harrison D. Collier‐Bain and Bruno Spellanzon; Menstrual cycle section written by Alannah K. A. McKay, Jennifer Maher, Louise Bradshaw and Rachel Eddy; Timing section written by James A. Betts, Harry A. Smith and Daniel Rees. Reviewing and editing of final version by Lucy H. Merrell, Oliver J. Perkin, Francoise Koumanov, Dylan Thompson, Javier T. Gonzalez and James A. Betts. All authors have read and approved the final version of this manuscript and agree to be accountable for all aspects of the work in ensuring that questions related to the accuracy or integrity of any part of the work are appropriately investigated and resolved. All persons designated as authors qualify for authorship, and all those who qualify for authorship are listed.

## CONFLICT OF INTEREST

A.K.A.M. has received research funding from the Australian Institute of Sport, Boston Children's Hospital's Female Athlete Program, Amazentis Life Sciences and the Swiss National Science Foundation; and has completed paid consultancy for PepsiCo. L.J.J. has current/previous funding from Entrinsic Beverage Company LLC, Entrinsic Bioscience LLC, Herbalife Europe Ltd, Bridge Farm Nurseries, Decathlon SA, PepsiCo Inc., Volac International; has performed consultancy for PepsiCo Inc. and Lucozade Ribena Suntory; and has received conference fees from PepsiCo Inc. and Danone Nutricia. In all cases, monies have been paid to L.J.J.’s institution and not directly to L.J.J. J.T.G. has received research funding from BBSRC, MRC, British Heart Foundation, Clasado Biosciences, Lucozade Ribena Suntory, ARLA Foods Ingredients and Cosun Nutrition Center; is a scientific advisory board member to ZOE and 6d Sports Nutrition; and has completed paid consultancy for The Dairy Council, PepsiCo, Violicom Medical, Tour Racing Ltd, and SVGC. J.A.B. is a named investigator on research grants funded by BBSRC, MRC, British Heart Foundation, Rare Disease Foundation, EU Hydration Institute, GlaxoSmithKline, Nestlé, Lucozade Ribena Suntory, ARLA foods, Cosun Nutrition Center, American Academy of Sleep Medicine Foundation and Salus Optima (L3M Technologies Ltd); has completed paid consultancy for PepsiCo, Kellogg's, SVGC and Salus Optima (L3M Technologies Ltd); is Company Director of Metabolic Solutions Ltd; receives an annual honorarium as a member of the academic advisory board for the International Olympic Committee Diploma in Sports Nutrition; and receives an annual stipend as Editor‐in Chief of *International Journal of Sport Nutrition & Exercise Metabolism*.

## FUNDING INFORMATION

None.

## References

[eph13497-bib-0001] Altman, D. G. , & Bland, J. M. (2005). Treatment allocation by minimisation. British Medical Journal, 330(7495), 843. 15817555 10.1136/bmj.330.7495.843 PMC556084

[eph13497-bib-0002] Arana Echarri, A. , Struszczak, L. , Beresford, M. , Campbell, J. P. , Jones, R. H. , Thompson, D. , & Turner, J. E. (2023). Immune cell status, cardiorespiratory fitness and body composition among breast cancer survivors and healthy women: A cross sectional study. Frontiers in Physiology, 14, 1107070. 37324393 10.3389/fphys.2023.1107070 PMC10267418

[eph13497-bib-0003] Atherton, P. J. , Etheridge, T. , Watt, P. W. , Wilkinson, D. , Selby, A. , Rankin, D. , Smith, K. , & Rennie, M. J. (2010). Muscle full effect after oral protein time‐dependent concordance and discordance between human muscle protein synthesis and mTORC1 signaling. American Journal of Clinical Nutrition, 92(5), 1080–1088. 20844073 10.3945/ajcn.2010.29819

[eph13497-bib-0004] Baker, F. C. , & Driver, H. S. (2007). Circadian rhythms, sleep, and the menstrual cycle. Sleep Medicine, 8(6), 613–622. 17383933 10.1016/j.sleep.2006.09.011

[eph13497-bib-0005] Bardis, C. N. , Kavouras, S. A. , Kosti, L. , Markousi, M. , & Sidossis, L. S. (2013). Mild hypohydration decreases cycling performance in the heat. Medicine and Science in Sports and Exercise, 45(9), 1782–1789. 23470313 10.1249/MSS.0b013e31828e1e77

[eph13497-bib-0006] Bateman, L. , McSwain, R. , Lott, T. , Brown, T. , Cemenja, S. , Jenkins, J. , Tapper, A. , Parr, J. , & Dolbow, D. (2023). Effects of Ibuprofen on muscle hypertrophy and inflammation: A review of literature. Current Physical Medicine and Rehabilitation Reports, 11(1), 43–50.

[eph13497-bib-0007] Bates, B. , Lennox, A. , Prentice, A. , Bates, A. , Page, P. , Nicholson, S. , & Swan, G. (2014). National Diet and Nutrition Survey: Results from Years 1, 2, 3 and 4 (combined) of the Rolling Programme (2008/2009 –2011/2012). Retrieved from https://assets.publishing.service.gov.uk/media/5a80dbd840f0b62302695e6d/NDNS_Y1_to_4_UK_report_executive_summary_revised_February_2017.pdf

[eph13497-bib-0008] Benton, M. J. , Hutchins, A. M. , & Dawes, J. J. (2020). Effect of menstrual cycle on resting metabolism: A systematic review and meta‐analysis. PLoS ONE, 15(7), e0236025. 32658929 10.1371/journal.pone.0236025 PMC7357764

[eph13497-bib-0009] Bone, J. L. , Ross, M. L. , Tomcik, K. A. , Jeacocke, N. A. , Hopkins, W. G. , & Burke, L. M. (2017). Manipulation of muscle creatine and glycogen changes dual x‐ray absorptiometry estimates of body composition. Medicine and Science in Sports and Exercise, 49(5), 1029–1035. 28410328 10.1249/MSS.0000000000001174

[eph13497-bib-0010] Cheuvront, S. N. , Kenefick, R. W. , & Zambraski, E. J. (2015). Spot urine concentrations should not be used for hydration assessment: A methodology review. International Journal of Sport Nutrition and Exercise Metabolism, 25(3), 293–297. 25386829 10.1123/ijsnem.2014-0138

[eph13497-bib-0011] Colenso‐Semple, L. M. , D'Souza, A. C. , Elliott‐Sale, K. J. , & Phillips, S. M. (2023). Current evidence shows no influence of women's menstrual cycle phase on acute strength performance or adaptations to resistance exercise training [Systematic Review]. Frontiers in Sports and Active Living, 5, 1054542. 37033884 10.3389/fspor.2023.1054542 PMC10076834

[eph13497-bib-0012] El‐Chab, A. , Simpson, C. , & Lightowler, H. (2016). The reproducibility of a diet using three different dietary standardisation techniques in athletes. European Journal of Clinical Nutrition, 70(8), 954–958. 27094626 10.1038/ejcn.2016.55

[eph13497-bib-0013] El‐Chab, A. , Simpson, C. , & Lightowler, H. (2019). The effect of consuming a liquid diet vs a solid diet 24‐hr preexperimental trials on adherence in athletes. International Journal of Sport Nutrition and Exercise Metabolism, 29(5), 493–497. 30676140 10.1123/ijsnem.2018-0259

[eph13497-bib-0014] Elliott‐Sale, K. J. , Minahan, C. L. , de Jonge, X. , Ackerman, K. E. , Sipilä, S. , Constantini, N. W. , Lebrun, C. M. , & Hackney, A. C. (2021). Methodological considerations for studies in sport and exercise science with women as participants: A working guide for standards of practice for research on women. Sports Medicine, 51(5), 843–861. 33725341 10.1007/s40279-021-01435-8 PMC8053180

[eph13497-bib-0015] Gill, J. M. , & Hardman, A. E. (2000). Postprandial lipemia: Effects of exercise and restriction of energy intake compared. American Journal of Clinical Nutrition, 71(2), 465–471. 10648259 10.1093/ajcn/71.2.465

[eph13497-bib-0016] Gillette, C. A. , Bullough, R. C. , & Melby, C. L. (1994). Postexercise energy expenditure in response to acute aerobic or resistive exercise. International Journal of Sport Nutrition, 4(4), 347–360. 7874151 10.1123/ijsn.4.4.347

[eph13497-bib-0017] Horowitz, J. F. (2007). Exercise‐induced alterations in muscle lipid metabolism improve insulin sensitivity. Exercise and Sport Sciences Reviews, 35(4), 192–196. 17921788 10.1097/jes.0b013e318156e084

[eph13497-bib-0018] Janse, D. E. J. X. , Thompson, B. , & Han, A. (2019). Methodological recommendations for menstrual cycle research in sports and exercise. Medicine and Science in Sports and Exercise, 51(12), 2610–2617. 31246715 10.1249/MSS.0000000000002073

[eph13497-bib-0019] Jeacocke, N. A. , & Burke, L. M. (2010). Methods to standardize dietary intake before performance testing. International Journal of Sport Nutrition and Exercise Metabolism, 20(2), 87–103. 20479482 10.1123/ijsnem.20.2.87

[eph13497-bib-0020] Jensen, J. , Rustad, P. I. , Kolnes, A. J. , & Lai, Y. C. (2011). The role of skeletal muscle glycogen breakdown for regulation of insulin sensitivity by exercise. Frontiers in Physiology, 2, 112. 22232606 10.3389/fphys.2011.00112 PMC3248697

[eph13497-bib-0021] Juliano, L. M. , & Griffiths, R. R. (2004). A critical review of caffeine withdrawal: Empirical validation of symptoms and signs, incidence, severity, and associated features. Psychopharmacology, 176(1), 1–29. 15448977 10.1007/s00213-004-2000-x

[eph13497-bib-0022] Juntip, N. , & Pornpimol, M. (2021). Familiarization effects of five‐time sit to stand, timed up and go, and six‐minute walk test in healthy young and older adult. Journal of Exercise Physiology online, 24(6), 77–86.

[eph13497-bib-0023] Kim, I. Y. , Park, S. , Chou, T. H. , Trombold, J. R. , & Coyle, E. F. (2016). Prolonged sitting negatively affects the postprandial plasma triglyceride‐lowering effect of acute exercise. American Journal of Physiology‐Endocrinology and Metabolism, 311(5), E891–E898. 27702747 10.1152/ajpendo.00287.2016

[eph13497-bib-0024] Kozior, M. , Jakeman, P. M. , & Norton, C. (2022). Dietary standardisation in a nutrient plus exercise intervention: Derivation, implementation, and evaluation. Journal of Food and Nutrition Research, 10(7), 488–495.

[eph13497-bib-0025] Logan‐Sprenger, H. M. , Heigenhauser, G. J. , Jones, G. L. , & Spriet, L. L. (2015). The effect of dehydration on muscle metabolism and time trial performance during prolonged cycling in males. Physiological Reports, 3(8), e12483. 26296770 10.14814/phy2.12483 PMC4562569

[eph13497-bib-0026] Lundberg, T. R. , & Howatson, G. (2018). Analgesic and anti‐inflammatory drugs in sports: Implications for exercise performance and training adaptations. Scandinavian Journal of Medicine & Science in Sports, 28(11), 2252–2262. 30102811 10.1111/sms.13275

[eph13497-bib-0027] Maseda, D. , & Ricciotti, E. (2020). NSAID‐gut microbiota interactions. Frontiers in Pharmacology, 11, 1153. 32848762 10.3389/fphar.2020.01153 PMC7426480

[eph13497-bib-0028] Mattocks, K. T. , Buckner, S. L. , Jessee, M. B. , Dankel, S. J. , Mouser, J. G. , & Loenneke, J. P. (2017). Practicing the test produces strength equivalent to higher volume training. Medicine and Science in Sports and Exercise, 49(9), 1945–1954. 28463902 10.1249/MSS.0000000000001300

[eph13497-bib-0029] Mears, S. A. , Dickinson, K. , Bergin‐Taylor, K. , Dee, R. , Kay, J. , & James, L. J. (2018). Perception of breakfast ingestion enhances high‐intensity cycling performance. International Journal of Sports Physiology and Performance, 13(4), 504–509. 28952831 10.1123/ijspp.2017-0318

[eph13497-bib-0030] Meendering, J. R. , Torgrimson, B. N. , Houghton, B. L. , Halliwill, J. R. , & Minson, C. T. (2005). Menstrual cycle and sex affect hemodynamic responses to combined orthostatic and heat stress. American Journal of Physiology‐Heart and Circulatory Physiology, 289(2), H631–H642. 15778279 10.1152/ajpheart.00029.2005

[eph13497-bib-0031] Morin‐Papunen, L. , Martikainen, H. , McCarthy, M. I. , Franks, S. , Sovio, U. , Hartikainen, A. L. , Ruokonen, A. , Leinonen, M. , Laitinen, J. , Järvelin, M. R. , & Pouta, A. (2008). Comparison of metabolic and inflammatory outcomes in women who used oral contraceptives and the levonorgestrel‐releasing intrauterine device in a general population. American Journal of Obstetrics and Gynecology, 199(5), 529.e1–529.e10. 10.1016/j.ajog.2008.04.013 18533124

[eph13497-bib-0032] Naharudin, M. N. , Adams, J. , Richardson, H. , Thomson, T. , Oxinou, C. , Marshall, C. , Clayton, D. J. , Mears, S. A. , Yusof, A. , Hulston, C. J. , & James, L. J. (2020). Viscous placebo and carbohydrate breakfasts similarly decrease appetite and increase resistance exercise performance compared with a control breakfast in trained males. British Journal of Nutrition, 124(2), 232–240. 10.1017/S0007114520001002 32174286

[eph13497-bib-0033] Oosthuyse, T. , & Bosch, A. N. (2010). The effect of the menstrual cycle on exercise metabolism: implications for exercise performance in eumenorrhoeic women. Sports Medicine, 40(3), 207–227. 20199120 10.2165/11317090-000000000-00000

[eph13497-bib-0034] Palmisano, B. T. , Zhu, L. , Eckel, R. H. , & Stafford, J. M. (2018). Sex differences in lipid and lipoprotein metabolism. Molecular Metabolism, 15, 45–55. 29858147 10.1016/j.molmet.2018.05.008 PMC6066747

[eph13497-bib-0035] Piltonen, T. , Puurunen, J. , Hedberg, P. , Ruokonen, A. , Mutt, S. J. , Herzig, K. H. , Nissinen, A. , Morin‐Papunen, L. , & Tapanainen, J. S. (2012). Oral, transdermal and vaginal combined contraceptives induce an increase in markers of chronic inflammation and impair insulin sensitivity in young healthy normal‐weight women: a randomized study. Human Reproduction, 27(10), 3046–3056. 22811306 10.1093/humrep/des225

[eph13497-bib-0036] Poggiogalle, E. , Jamshed, H. , & Peterson, C. M. (2018). Circadian regulation of glucose, lipid, and energy metabolism in humans. Metabolism, 84, 11–27. 29195759 10.1016/j.metabol.2017.11.017 PMC5995632

[eph13497-bib-0037] Rose, G. A. , Davies, R. G. , Davison, G. W. , Adams, R. A. , Williams, I. M. , Lewis, M. H. , Appadurai, I. R. , & Bailey, D. M. (2018). The cardiopulmonary exercise test grey zone; optimising fitness stratification by application of critical difference. British Journal of Anaesthesia, 120(6), 1187–1194. 29793585 10.1016/j.bja.2018.02.062

[eph13497-bib-0038] Ruge, T. , Hodson, L. , Cheeseman, J. , Dennis, A. L. , Fielding, B. A. , Humphreys, S. M. , Frayn, K. N. , & Karpe, F. (2009). Fasted to fed trafficking of Fatty acids in human adipose tissue reveals a novel regulatory step for enhanced fat storage. Journal of Clinical Endocrinology and Metabolism, 94(5), 1781–1788. 19223522 10.1210/jc.2008-2090

[eph13497-bib-0039] Schutz, Y. (2000). Role of substrate utilization and thermogenesis on body‐weight control with particular reference to alcohol. Proceedings of the Nutrition Society, 59(4), 511–517. 11115785 10.1017/s0029665100000744

[eph13497-bib-0040] Smith, H. A. , & Betts, J. A. (2022). Nutrient timing and metabolic regulation. The Journal of Physiology, 600(6), 1299–1312. 35038774 10.1113/JP280756 PMC9305539

[eph13497-bib-0041] Starling, R. D. , Trappe, T. A. , Parcell, A. C. , Kerr, C. G. , Fink, W. J. , & Costill, D. L. (1997). Effects of diet on muscle triglyceride and endurance performance. Journal of Applied Physiology (1985), 82(4), 1185–1189. 10.1152/jappl.1997.82.4.1185 9104855

[eph13497-bib-0042] Subar, A. F. , Freedman, L. S. , Tooze, J. A. , Kirkpatrick, S. I. , Boushey, C. , Neuhouser, M. L. , Thompson, F. E. , Potischman, N. , Guenther, P. M. , Tarasuk, V. , Reedy, J. , & Krebs‐Smith, S. M. (2015). Addressing current criticism regarding the value of self‐report dietary data. Journal of Nutrition, 145(12), 2639–2645. 26468491 10.3945/jn.115.219634 PMC4656907

[eph13497-bib-0043] Thompson, D. , & Batterham, A. M. (2013). Towards integrated physical activity profiling. PLoS ONE, 8(2), e56427. 23437131 10.1371/journal.pone.0056427 PMC3577906

[eph13497-bib-0044] Turner, J. E. , Aldred, S. , Witard, O. C. , Drayson, M. T. , Moss, P. M. , & Bosch, J. A. (2010). Latent cytomegalovirus infection amplifies CD8 T‐lymphocyte mobilisation and egress in response to exercise. Brain, Behavior, and Immunity, 24(8), 1362–1370. 20638470 10.1016/j.bbi.2010.07.239

[eph13497-bib-0045] Zaura, E. , Brandt, B. W. , Teixeira de Mattos, M. J. , Buijs, M. J. , Caspers, M. P. , Rashid, M. U. , Weintraub, A. , Nord, C. E. , Savell, A. , Hu, Y. , Coates, A. R. , Hubank, M. , Spratt, D. A. , Wilson, M. , Keijser, B. J. , & Crielaard, W. (2015). Same exposure but two radically different responses to antibiotics: Resilience of the salivary microbiome versus long‐term microbial shifts in feces. Microbiology, 6(6), e01693‐01615. 10.1128/mBio.01693-15 PMC4659469 26556275

